# Sexual Dimorphism in the Response of *Mercurialis annua* to Stress

**DOI:** 10.3390/metabo6020013

**Published:** 2016-04-26

**Authors:** Ezra M. Orlofsky, Giorgi Kozhoridze, Lyudmila Lyubenova, Elena Ostrozhenkova, J. Barbro Winkler, Peter Schröder, Adelbert Bacher, Wolfgang Eisenreich, Micha Guy, Avi Golan-Goldhirsh

**Affiliations:** 1 French Associates Institute for Agriculture and Biotechnology of Drylands, Jacob Blaustein Institutes for Desert Research, Ben-Gurion University of the Negev, Midreshet Ben-Gurion 8499000, Israel; orlofsky@bgu.ac.il (E.M.O.); kozhoridze@gmx.com (G.K.); michagu@bgu.ac.il (M.G.); 2 Environmental Genomics Research Unit, Helmholtz Zentrum München, Deutsches Forschungszentrum für Gesundheit und Umwelt (GmbH), Neuherberg 85764, Germany; lyudmila.lyubenova@gmail.com (L.L.); peter.schroeder@helmholtz-muenchen.de (P.S.); 3 Lehrstuhl für Biochemie, Technische Universität München, Garching 85748, Germany; elena.ostrozhenkova@gmail.com (E.O.); adelbert.bacher@t-online.de (A.B.); wolfgang.eisenreich@mytum.de (W.E.); 4 Environmental Simulation Research Unit, Institute of Biochemical Plant Pathology, Helmholtz Zentrum München, Deutsches Forschungszentrum für Gesundheit und Umwelt (GmbH), Neuherberg 85764, Germany; bwinkler@helmholtz-muenchen.de (J.B.W.)

**Keywords:** anti-oxidation, dioecious, metabolism, salinity, senescence, stress

## Abstract

The research presented stemmed from the observations that female plants of the annual dioecious *Mercurialis annua* outlive male plants. This led to the hypothesis that female plants of *M. annua* would be more tolerant to stress than male plants. This hypothesis was addressed in a comprehensive way, by comparing morphological, biochemical and metabolomics changes in female and male plants during their development and under salinity. There were practically no differences between the genders in vegetative development and physiological parameters. However, under salinity conditions, female plants produced significantly more new reproductive nodes. Gender-linked differences in peroxidase (POD) and glutathione transferases (GSTs) were involved in anti-oxidation, detoxification and developmental processes in *M. annua*. ^1^H NMR metabolite profiling of female and male *M. annua* plants showed that under salinity the activity of the TCA cycle increased. There was also an increase in betaine in both genders, which may be explainable by its osmo-compatible function under salinity. The concentration of ten metabolites changed in both genders, while ‘Female-only-response’ to salinity was detected for five metabolites. In conclusion, dimorphic responses of *M. annua* plant genders to stress may be attributed to female plants’ capacity to survive and complete the reproductive life cycle.

## 1. Introduction

Plants, as sessile organisms, encounter environmental conditions that play a key role in their evolutionary strategies for survival. Extreme environmental conditions were conducive for development of adaptive tolerance mechanisms, including diverse reproductive systems. It was suggested that the evolution of dioecy, where female and male functions are born on separate individuals, enables better capacity to cope with limited resources in stressful environmental conditions [1]. A comprehensive review on the relationship between dioecious plants and stress tolerance was recently published by Juvany and Munné-Bosch (2015) [2].

Many of the stresses faced by living organisms involve the formation of reactive oxygen species (ROS), which are innate to life under oxygenic atmosphere. ROS are formed by normal metabolism, but their formation is enhanced under abiotic and biotic stresses such as: drought, salinity, developmental and stress induced senescence, herbivores and pathogens [3,4,5]. Plants respond to the stresses by induction of anti-oxidative defenses (for a review see Foyer and Noctor, 2009) [6], such as increased activity of anti-oxidative isoenzymes and anti-oxidants [7,8,9]. These activities are well orchestrated, with SOD isoenzymes functioning first in the conversion of the highly reactive superoxide radical into H_2_O_2_, a less harmful molecule that is detoxified by various peroxidases, of which APX is prominent [4]. At low concentrations, hydrogen peroxide can also serve as a signaling molecule that participates in stress responses, growth and development [4]. In addition, anti-oxidant metabolites such as the water-soluble ascorbate (ASC) and glutathione [10] and the lipophilic tocopherols [11] constitute part of the anti-oxidative machinery. It has been reported that glutathione transferases (GSTs) may also contribute to alleviation of oxidative stress [4].

Changes in plant metabolism reflect the appropriate adjustments needed to cope with various stresses and may hold a clue to the metabolic pathways most affected by stress [12,13]. Recently, high-resolution NMR has become an important technology for the elucidation of biosynthetic pathways and metabolite fluxes in plants under normal and stress conditions via quantitative assessment of metabolite and/or ^13^C-isotopologue profiles (for review, see Eisenreich and Bacher, 2007) [14]. Metabolomics is still used scantily for studies on gender related metabolism [15,16,17].

**Figure 1:**
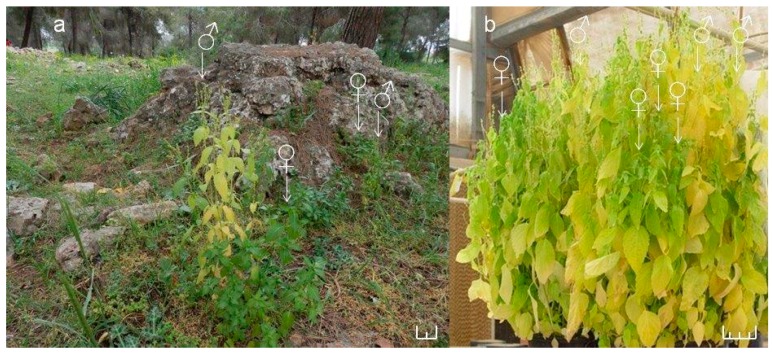
***Mercurialis annua*** female and male plants in nature and controlled conditions. Plants in nature in the Lower Galilee (32°43′08′′ N 35°23′38′′ E) (a) and grown densely under controlled conditions at ‘late senescence’ stage (b). Female *M. annua* plants bear sub-sessile flowers, being borne on short pedicels in the leaf axils. Male plants are morphologically distinct, with flowers on longpedunculated inflorescences. The scale bar represents 5 cm.

The annual, dioecious *Mercurialis annua* (Euphorbiaceae), used in this study is a common roadside herb native to the Mediterranean basin, which has spread into Europe, North America and Australia [18,19]. The diploid (2*n* = 16) *M. annua* species is a strictly dioecious annual, while the polyploid species are not [20]. The small annual plant has a short life cycle of approximately 6 weeks from seed to seed and indeterminate growth up to senescence after approx. 4 months. Before flowering, female and male *M. annua* plants are indistinguishable and can be identified by gender specific molecular markers [21,22,23]. In the indeterminate growth of *M. annua*, female plants keep producing seeds until late senescence, while the male plants enter the senescence stage before the female plants as seen in nature (Figure 1a). A similar phenomenon was observed under experimental conditions when seedlings were grown densely, competing for limited resources (Figure 1b). The apparent dimorphic resource allocation within and between female and male plants as well as the effect of various stresses on *M. annua* was critically addressed in several papers by Pannell’s group [24,25,26,27]. While the stress response of plants, in general, was extensively studied (reviewed in Zimmermann and Zentgraf, 2005) [28], the differential gender stress response is scantier. Dioecy has evolved in relatively a small number (6%) of plant species, mostly trees and a small number of annuals [29]. In this work we have resorted, experimentally, to the annual dioecious species, *Mercurialis annua*, that offers an advantageous system for studies on the dimorphic response of these plants to adverse environments.

Gender-specific-response to stresses was reported for some dioecious trees [30,31] and a few annual dioecious plants [32,33,34]. Developmental dimorphic response in flowering time and longevity were shown in the annual dioecious species: *Rumex hastatulus*, *Silene latifolia* and *Amaranthus cannabinus* [33,35,36,37]. These results pointed to the possibility that female and male plants possess different adaptabilities, which may be related to sex-specific responses under changing environments and to reproductive costs. In agreement to these reports, Case and Barrett (2004) [38] found that under aridity, a gynodioecious population of *Wurmbea dioica* exhibited a trend favoring a transition to dioecy. In contrast, Gehring and Linhart (1993) [39] found that females and males of dioecious *Silene latifolia* did not respond differently to low resources availability, although they did reaffirm that females allocate more resources needed to reproduction than do male plants [40]. These inconsistencies in the various studies are probably related to species and environmental differences. Therefore, it is important to increase the number of case studies and explore each case of dioecy on its own [41,42]. In this context, our observations that male plants exhibit stress-like sympthoms, such as yellowing, before female plants in *M. annua* (Figure 1), led us to the hypothesis that also under stress conditions, female *M. annua* plants would show higher tolerance than male plants. Annual dioecious plants provide an advantageous experimental model over dioecious perennials, they were hardly studied in relation to stresses. The effect of salinity stress in perennial dioecious species was reported for several species. Under salinity stress, *Amaranthus cannabinus* exhibited temporal gender segregation, where females displayed higher plasticity in flowering time as well as a longer growth period than males. It was concluded that greater reproductive efficiency, due to sex-specific growth patterns, might have been important in the evolution of this species [33]. Similarly, under changing photoperiods, in *Populus cathayana*, male leaves had a faster senescence than female leaves [43]. In *Ginkgo biloba*, female plants exhibited delayed senescence of autumn leaves, as compared to those of male plants [44]. The effect of senescence and salinity stresses on metabolism was reported for the dioecious Juniper [15] and poplar [17,30].

In this research, the dimorphic response of annual dioecious, *M. annua*, served as model system to test the phenotypic and metabolic response of female and male plants to salinity.

## 2. Results

### 2.1. Development of M. annua Plants under Control and in Response to Salinity

The growth of *M. annua* was operatively divided into five developmental stages, from the ‘young’ to the ‘late senescence’ stages (Table 1; Figure S1). These stages comprise: (a) ‘young’—at 55 days after sowing, when the plants had 5 nodes, and shoot height and the main root length were approximately 25 and 5 cm, respectively; (b) ’early maturity’—at 70 days; (c) ’mature’—at 84 days, when the plants became more branched and the leaves turned into dark green; (d) ‘early senescence’—at 98 days, when the plants were 60 cm in height, with 7 to 8 nodes and yellowing began to appear on male leaves, whereas in females it was delayed for approximately 3 weeks (Figure S1). (e) ’late senescence’—at 122 days, when both males and females were senescent. From ‘early maturity’ to ‘early senescence’ most of the growth resulted in stem extension with little root growth and new node formation (Table 1). The different speed of the senescence in both genders was expressed as a lower number of nodes in male plants (8) than in female (11) at ‘late senescence’ (Table 1). This increased number of nodes in female plants (that appears as a cluster at the top of the stem) occurred between 98 to 114 days, when male plants were already in the ‘late senescence’ stage. Plant growth is closely correlated with photosynthetic activity and is also reflected by several physiological parameters. Photosynthesis, stomatal conductance and transpiration rates of ‘young’ female and male plants did not differ significantly. Similarly, there was also no difference between the genders when exposed to salinity for a short term (2 h or 3 days) (Figure S2) or a long term (up to 59 days) (Figure S3).

Growth of mature female and male plants for 11 days in salinity had no effect on their stem lengths, root lengths or their fresh weights (Table 2). However, under salinity, female plants of *M. annua* had a higher number of nodes as compared to male plants and their controls (Table 2). The larger number of nodes in female plants was manifested as clustering of new nodes at the top of the stem and not due to stem extension (Figure S4).

Growth in the presence of salinity at the ‘mature stage’, for 11 days, led to some wilting and waxier leaves as compared to the leaves of untreated control plants (Figure S4c). In addition, leaves of NaCl-treated male plants appeared less greenish than female leaves, similar to the appearance of the senescent female and male leaves (Figure 1 and Figure S1f).

### 2.2. Anti-Oxidative and Detoxification Enzymes under Control and in Response to Salinity

**Figure 2:**
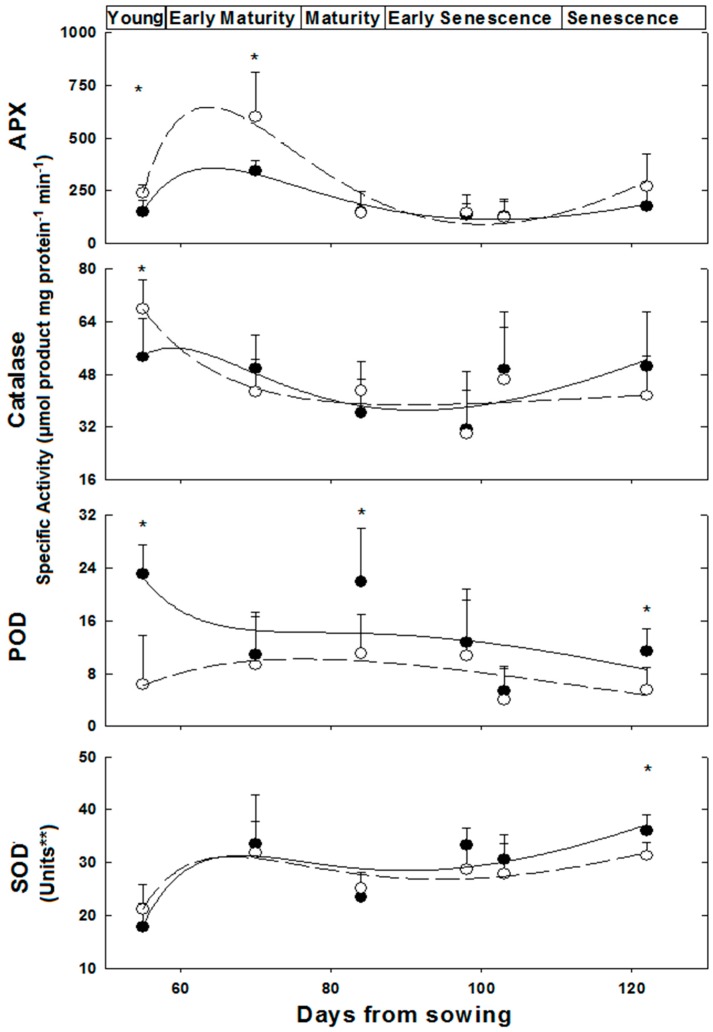
Anti-oxidative enzymes activity in *M. annua* female and male leaves during development. Plants were grown in soil without salt and at the indicated time points were harvested for analysis as indicated in the Experimental Section. The data was fitted by a 3rd order polynomial curve. Ave ± SD, *n* = 3. Open circle—female plant; Black circle—male plant. * indicate significant difference (*p* < 0.05) between female and male at a given day; ** One unit of SOD activity was defined as the amount of protein required to cause 50% inhibition of NBT reduction.

**Figure 3:**
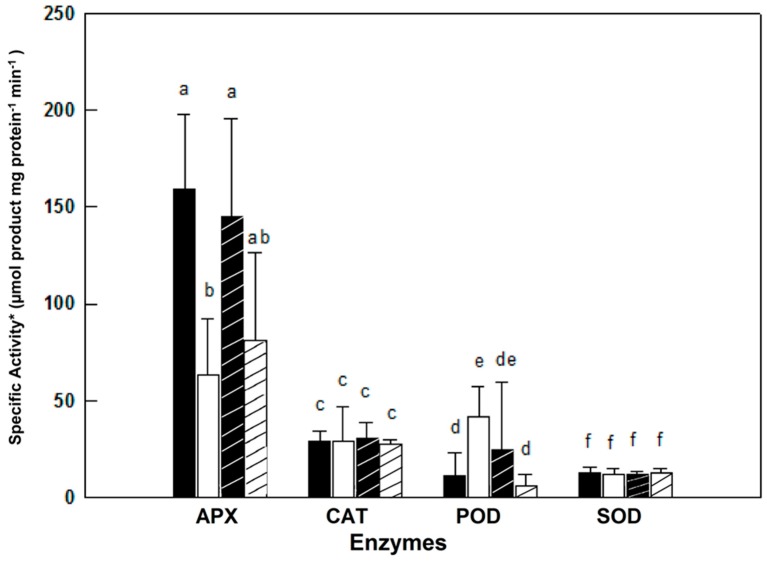
Effect of salinity on anti-oxidative enzymes activity in *M. annua* female and male leaves. ‘Mature stage’ plants were grown for 11 days in the presence or absence of NaCl (100 mM). The data was fitted by a 3rd order polynomial curve. Ave ± SD, *n* = 3. * One unit of SOD activity was defined as the amount of protein required to cause 50% inhibition of NBT reduction. Different letters for a given enzyme indicate grouping at a significant level of *p* < 0.05 by Anova-Fisher’s test. 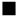
—female control; 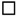
—male control; 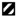
—female salinity; 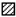
—male salinity.

Activities of the anti-oxidative isoenzymes APX, CAT, POD and SOD were determined along the growth of female and male plants from the ‘young’ to the ‘late senescence stages’ (Figure 2). The activities of these isoenzymes varied in leaves of female and male *M. annua* along the growth stages from day 55 to 122 (Figure 2). At ‘young’ and ‘early maturity’ stages, the female APX activity was significantly (*p* < 0.005) higher than the male activity. Both activities peaked at 70 days and then dropped continuously till reaching similar values between 84 and 103 days. At ‘late senescence’, activities of both genders increased (by 100% and 38% for female and male, respectively); yet, these later activities were not statistically different. Exposure of ‘mature’ (84 days) female and male plants to salinity did not affect their leaf APX activities (Figure 3). However, female leaf APX activity was approximately 2.3-fold higher than male under both control and salinity (Figure 3).

Catalase (CAT) activity in leaf of ‘young’ female *M. annua* was by 28% higher than that of male after that developmental phase, however, both male and female CAT activities dropped gradually and were not statistically different (Figure 2). Exposure of ‘mature’ female and male plants to salinity did not affect their leaf CAT activities (Figure 3).

Peroxidase (POD) activity was significantly higher in male plants at the ‘young’, ‘mature’ and ‘late senescence’ stages (Figure 2). Generally, male POD activity decreased linearly in the course of development, while in female plants, POD activity increased between the ‘young’ and ‘mature’ stages and gradually decreased towards ‘late senescence’. Leaf POD specific activity was substantially lower than those of APX and CAT. POD activity in salt treated male plants decreased significantly (*p* < 0.05) and female activity increased, however, not significantly (Figure 3).

A similar leaf SOD activity pattern was determined along the growth of female and male *M. annua* plants from the ‘young’ until the ‘late senescence’ stages (Figure 2). At day 70, SOD activity increased by 50% and decreased by 25% at day 84 for both genders, respectively. At ‘early senescence’ (103 days), same male and female SOD activity was found. However, at ’late senescence’ (122 days), SOD activity in the leaf of male plants was by 15% higher than that of the female (Figure 2). Exposure of ‘mature’ female and male plants to salinity did not affect their leaf SOD activities (Figure 3).

The ratios between the activities of each of the H_2_O_2_ leaf detoxification enzymes to that of SOD activity were calculated, where the ratio at the ‘young stage’ (55 days) was assigned a value of 1.0 (Figure S5). Generally, ratios lower than 1 were found for both genders, except for the APX/SOD ratio in female and male leaves at the ‘early maturity’ stages and for the POD/SOD ratio at ‘mature’ and ‘early senescence’ stages of female leaves (Figure S5).

Glutathione S-transferase (GST) activity in *M. annua* plants exposed to high salinity (150 mM NaCl) for a short time (48 h) was determined with model substrates. Whereas GST activity measured with the standard model substrates, CDNB and DCNB, did not significantly differ in salt treated and control plants, nor in plants of different gender (data not shown), the rates determined with the diphenylether herbicide, fluorodifen, as substrate were both sex and salt dependent (Table S1). The female leaf control had about five-fold higher fluorodifen-GST activity than male, while under salinity this activity was down-regulated in female, and up-regulated in male (Table S1). A similar trend was obtained in flowers (data not shown).

### 2.3. Content of Oxidative/Anti-Oxidative Substrates

**Figure 4:**
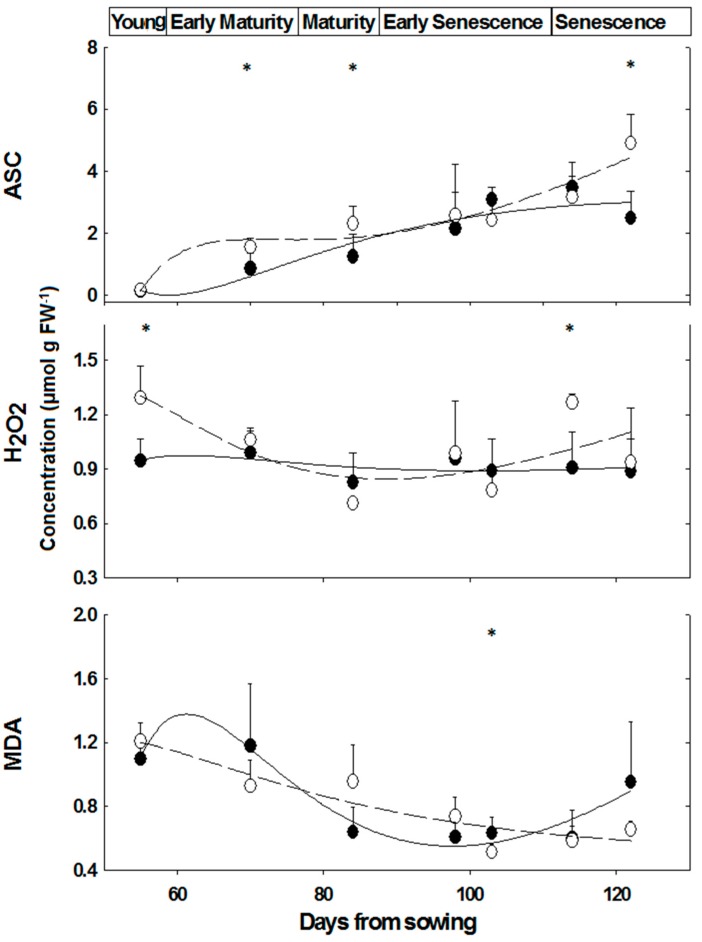
Ascorbate, hydrogen peroxide and MDA concentrations in leaves of *M. annua* female and male plants during development. Plants were grown in soil without salt and at the indicated time points were harvested for analysis as indicated in the Experimental Section. The data were fitted by a 3rd order polynomial curve. Ave ± SD, *n* = 3. Open circle—female plant; Black circle—male plant. * indicate significant difference (*p* < 0.05) between female and male at a given day.

Similar ASC contents were determined in leaves of ‘young’, while at ‘mature’ and ‘senescence’ stages, the difference between female and male *M. annua* plants was significant (*p* < 0.05) (Figure 4). ASC content, however, increased over time in both genders (Figure 4). At the ‘early senescence’ stage (98 days), female and male ASC contents were not significantly different (Figure 4). During senescence, ASC content increased in both genders, during the time interval between days 114 and 122, ASC content in females increased by 54%, while that of the male fell by 28% and as a result ASC content in male plants at day 122 was about half of that of the female’s (Figure 4).

**Figure 5:**
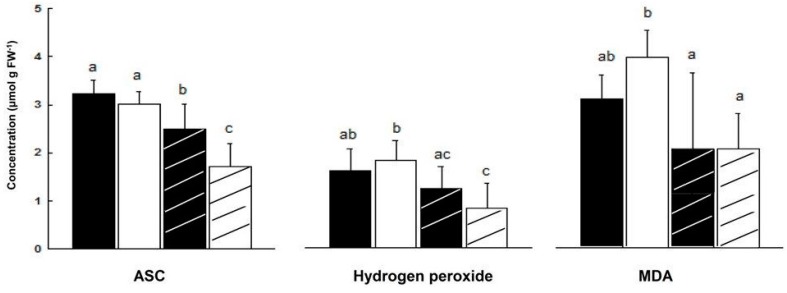
Effect of salinity on ASC, H_2_O_2_ and MDA concentrations in leaves of ‘mature’ female and male *M. annua* plants. The plants were grown for 11 days in the presence or absence of NaCl (100 mM). Ave ± SD, *n* = 3. Different letters for a given compound indicate grouping at a significant level of *p* < 0.05, by Anova-Fisher’s test. 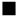
—female control; 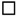
—male control; 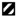
—female salinity; 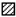
—male salinity.

In salt treated ‘mature’ plants (84 days), ASC leaf content decreased more in male than in female plants (44% and 22%, respectively). As a result, ASC content in leaves of NaCl treated female plants was higher by 47% than in leaves of NaCl treated male plants (Figure 5).

There was no significant difference in H_2_O_2_ content of male and female leaves during development, except at the ‘young’ and ‘senescence’ stages (Figure 4). In NaCl treated plants, H_2_O_2_ content fell in male leaves by 54% and by 25% in female leaves, although the decline in female leaf was not significant (Figure 5). Leaf hydrogen peroxide content of NaCl treated male plants was lower by 32% than that of female plants, however this difference was not significant (Figure 5).

During development, there was a trend of decline in leaf MDA content in female and male plants (Figure 4). In salt stressed plants, MDA content fell in male by 49% (*p* < 0.0005) and was lower by 67% in male than female leaves (2 and 3 μmol/g FW, respectively, *p* < 0.05, Figure 5).

### 2.4. Overall Metabolite Profiles of M. annua under Salinity

Using ^1^H-NMR, 29 major metabolites: amino acids (12), carbohydrates (4), TCA cycle compounds (6) and secondary metabolites (7) were identified in polar extracts of leaves and roots detached from ‘young’ (55 days) *M. annua* plants exposed to salinity for 3 days (Figure S6).

Multivariate analyses revealed that metabolite concentrations, of both genders under control and salinity, were significantly higher in leaves than in roots (*p* < 0.05), however, there was no significant difference between the total female and male plant metabolites concentration (*p* > 0.05, Table S2). No significant difference in metabolite concentrations was found between female and male leaves and roots, under salinity and control (*p* > 0.05, Table S2). Concentrations of amino acids, carbohydrates and TCA cycle metabolites were not significantly different between female and male plants (*p* > 0.05, Table S2), but the secondary metabolite concentrations were significantly (*p* < 0.05) higher in male plants (Table S2 and Figure S6). Metabolites concentrations in roots and leaves did not change significantly under salinity in a combined female and male analysis of the data. The effect of salinity on the concentration of various groups of metabolites, *i.e.*, amino acids, carbohydrates, TCA cycle metabolites and secondary metabolites in *M. annua* plants was not significant (Table S2).

**Figure 6:**
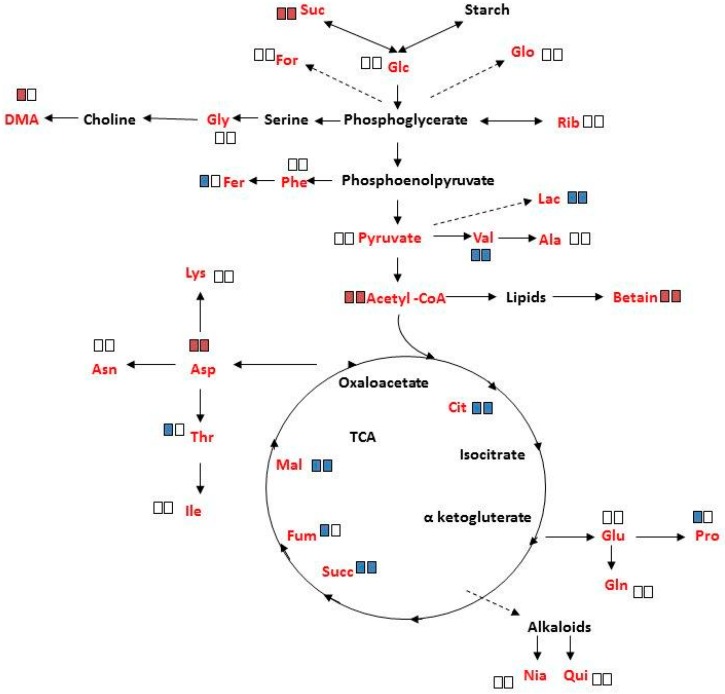
Schematic metabolic pathways showing changes in leaf metabolites concentration of ‘Young stage’ *M. annua* under salinity. Plant of *M. annua* at ‘young stage’ (55 days) were exposed to NaCl (100 mM) for 3 days. Extraction of polar metabolites from female and male leaf and root was done with perchloric acid. Left rectangle—female; right rectangle—male; red rectangle—increase; blue rectangle—decrease; Empty rectangle indicates no significant change under salinity. Significant changes at *p* < 0.05 are shown. Metabolites written in red were identified by ^1^H NMR. Metabolites written in black were below detection threshold.

The changes in specific metabolite concentrations under salinity treatment are shown in Figure 6 and Figure S6. In response to salinity, a significant increase (*p* < 0.05) was determined in sucrose, acetyl CoA, betaine and aspartate in mature female and male plants, with the exception that DMA had increased only in female plants. The decreased concentrations of lactate, valine, citrate, succinate and malate, under salinity, were common to both sexes. The decrease in ferulate, fumarate, threonine, and proline, under these conditions, was however unique to female plants. It should be noted that salinity did not significantly affect the concentration of the other measured metabolites.

## 3. Discussion

### 3.1. Dioecious Plant Development

The underlying hypothesis of this study was that female and male *M. annua* plants express dimorphic responses at the physiological, biochemical and metabolic levels, under stress and non-stress conditions. There were no significant differences, until early senescence, between female and male plants in the vegetative parameters, *i.e.*, stem and root lengths, photosynthesis, transpiration and stomatal conductance. Differences, however, were distinct at senescence and under salinity (Figure 1 and Figure S4 & Table 1 and Table 2). In *M. annua*, a significantly higher (*p* < 0.05) number of reproductive nodes characterized senescing female plants in comparison to male plants (Table 1). Similarly, salinity-treated mature female plants exhibited significantly higher number of reproductive nodes than male plants (Table 2), suggesting that female plants could direct resources towards reproductive development for securing completion of the reproductive life cycle and seed production. It has been reported already at the early 20th century that senescing male plants of the annual dioecious, *Cannabis sativa*, *Trinia glauca* (L.) Dumort. and *Spinacia oleracea* die earlier than female plants [43]. This was attributed to a difference in the reproductive roles of the sexes, male functions end with pollen shedding, while females must ripen fruits. Indeed, dioecious plants were shown to exhibit gender dependent onset of senescence, for example in *Populus cathayana* and *Ginkgo biloba,* male leaves were earlier senescent than female leaves [44,45].

### 3.2. Enzymes and Substrates during Development and Salinity

The developmental shift, in female plants towards higher number of reproductive nodes at the late growth stage, *i.e.*, senescence and under salinity (Table 1 and Table 2) led to further research on accompanying metabolic processes in *M. annua*. These conditions are characterized by metabolic changes and enhanced ROS production, respectively [5,28]. Among the anti-oxidative enzymes, APX and catalase activity were higher in female plants at early developmental stages, while SOD activity was significantly higher in male plants at ‘late senescence’ (Figure 2). Cellular hydrogen peroxide content is mostly determined by the interplay between its synthesis via SOD isoenzymes and detoxification rates catalyzed by the various peroxidase-like isoenzymes and catalase. These enzymes are localized in various cell organelles and the cytoplasm, where the cellular distribution of H_2_O_2_ is mediated by aquaporin. In addition, soluble cytoplasmic and organellar anti-oxidants such as ascorbate and glutathione participate in its detoxification [10]. Hydrogen peroxide content was fairly similar in female and male plants and stable along the growth of *M. annua* plants, except at the ‘young’ developmental stage, when higher concentration was measured in female leaves (Figure 4), in spite of a higher than one APX to SOD ratio (Figure S5). The difficulty to explain the above discrepancy might be supported by the lack of significant differences in MDA between female and male plants, in most of the developmental course, and a general trend of decline in MDA concentration during development (Figure 4). MDA is a membrane lipid peroxidation product and is associated with oxidative damage [46]. Taken together, a plausible explanation is that the higher content of H_2_O_2_ serves in ‘young’ female plants in signaling rather than as an oxidant [5,6].

Components of the anti-oxidative system are multifunctional, in defense as well as in developmental and signaling processes [6]. For example, in both genders, POD and CAT specific activities were significantly lower (by at least 10-fold) than that of APX (Figure 2). This may indicate that POD activity plays a role in development rather than in anti-oxidative protection *per se*, in agreement with Asada’s (1992) [47] classification of POD as primarily involved in physiological processes distinct from H_2_O_2_ scavenging. Furthermore, the higher POD activity in male plants, throughout development (Figure 2), may be associated with the earlier initiation of senescence in that gender. Worth noting is that higher POD activity was also reported in senescing pea leaves [48].

At ‘young’ and ‘early maturity’, female APX activity was higher as compared to males, and declined significantly in later developmental stages (Figure 2). This decline in APX activity, and the resulting lower H_2_O_2_ detoxification capacity may be compensated by a concomitant increase in ASC concentration; accordingly, the slight increase that was observed in females, peaking at ‘late senescence’, added to their potential to detoxify hydrogen peroxide (Figure 4).

It was assumed that challenging *M. annua* genders with salinity would yield a gender specific response similar to the developmental senescence response. Leaf chlorosis, the first noticeable indicator of senescence-associated programmed cell death and a well-known consequence of ROS accumulation [49] was apparent in *M. annua* under senescence and salinity (Figure 1 and Figure S4). Growth of female and male *M. annua* plants under salinity did not indeed affect the anti-oxidative enzymes, APX, CAT and SOD (Figure 3). However, an APX gender-dependent activity was found that was significantly higher in female compared to male plants. It should be noted that overall POD activity was much lower than that of APX and that under salinity only male POD activity was significantly decreased, indicating that like under senescence, POD activity might be related to processes other than H_2_O_2_ detoxification [47]. While anti-oxidative enzyme activities were largely unaffected by salinity (Figure 3), endogenous ASC and H_2_O_2_ contents decreased significantly, compared to their respective controls, more so in the male than in the female (Figure 5). The decline in ASC concentration in salt treated male leaves was correlated to its use in the reduction (dual meaning) of H_2_O_2_ by approx. 50% (Figure 5) and may reflect lower regeneration capacity under these conditions. Like in senescence, MDA concentration in female leaves was not significantly affected by salinity (Figure 5), suggesting that their membranes were not damaged. The finding that MDA concentration in males decreased under salinity cannot be explained at present. Preferential salt-induced lipid peroxides accumulation was reported in several salt-sensitive species, but not in their salt-tolerant relatives [6,50]. Thus, the finding that in *M. annua* there was not accumulation of MDA under salinity (Figure 5), taken together with the finding that vegetative growth was not affected by salinity (Table 2), may suggest that *M. annua* is not a salt-sensitive species.

The GST-superfamily isoenzymes participate in various cellular developmental processes and in stress tolerance in plants [51]. It was shown here, for the first time, that in the dioecious *M. annua*, GSTs are differentially expressed in female and male plants at early senescence and under salinity (Table S1). It is noteworthy that also in animals a gender-linked difference in GST expression under toxic conditions was reported [52,53]. In contrast, at early senescence APX, CAT and SOD activities were similar in both genders (Figure 2). Similarly to POD, GSTs may also participate in regulatory and developmental processes, as well as detoxification reactions under stress [51]. In accordance, the higher GST activity of control female plants may be linked to differential occurrence of GST isoforms [54]. This might be also correlated to continued formation of new reproductive nodes and protection against accumulation of toxic intermediates and may explain the longer lifespan of female *M. annua* plants (Figure 1 and Figure S4).

### 3.3. Metabolite Changes in M. annua under Salinity

Unbiased methods of “metabolomics” were used to study the effect of salt stress on grapevines [55], *Arabidopsis thaliana* [49], rice [54], and others. More recently, the effect of age and salinity on dioecious Juniper [16] and poplar [16,17] metabolism was reported. The use of ^1^H NMR for investigation of metabolite compositions from Cd-stressed *Silene cucubalus* [56,57] and salt-stressed tobacco plants [58] was reported recently. A literature survey revealed that the effect of salinity on the metabolome of annual dioecious species has not been reported. A recently published research [59], comparing metabolites of medicinal interest in *M. annua* and *M. perennis*, in the context of species classification, was reported. However, gender related difference in the metabolites was not in the scope of that research.

In our study, we took advantage of ^1^H NMR to analyze metabolite mixtures without prior analytical separation of crude extracts and compared NMR data of salinity-treated female and male *M. annua* plants in comparison to control plants (Table S2; Figure 6).

Analysis of variance of metabolite concentrations in female and male *M. annua*, under control and salinity treatments, showed no statistical difference (Table S2). Under salinity, however, the concentration of ten metabolites changed (either increased or decreased) in both genders. ‘Female-only-response’ to salinity was detected for five metabolites, of which only dimethylamine (DMA) concentration increased, while that of ferulate, threonine, fumarate and proline decreased (Figure 6). These results suggested more responsiveness of female *M. annua* to the salinity. In both genders there was an increase in acetyl CoA and oxaloacetate entering the TCA cycle. It is tempting to speculate that fluxes *via* glycolysis and the TCA were lowered in response to salinity leading to lower amounts of e.g., lactate and citrate. In agreement, more free sucrose is found under salinity conditions. The increased amounts of acetyl CoA could then indicate increased lipolysis and β-oxidation of fatty acids (maybe to provide FADH_2_ and NADH thereby compensating the decreasing TCA activity). The increase in betaine in both female and male plants may be explainable by its osmo-compatible function under salinity. However, the decrease in proline in female only and the fact that the concentration of both compounds was very low may suggest that they do not function as a compatible solute in *M. annua* or that the short term of exposure to salinity was not enough for significant concentration build-up of these compounds (Figure 6; Figure S6). These results are in contrast to what was reported for *Populus yunnanensis*, where female plants were less tolerant to salinity than male plants [16,30]. These distinctive responses may reflect also differences between annual and perennial plants.

In conclusion, *M. annua* female plants are more tolerant to salinity and exhibit longer lifespan than the male. We could show that the anti-oxidative response was fairly similar in female and male plants. The better female performance under senescence and salinity was correlated with a more active APX and higher ascorbate concentration. It appears that the multifunctional characteristics of POD and GSTs were involved in anti-oxidation, detoxification and developmental processes in *M. annua*. Moreover, major gender-linked differences in these enzymes, in addition to female-specific metabolite concentration changes, may have attributed to female plants superior regulatory adjustment capacity, longer survival and securing completion of the reproductive life cycle.

## 4. Experimental Section

### 4.1. Plant Material and Growth Conditions

Seeds of *Mercurialis annua* of Belgian origin, collected near the University of Ghent and kindly provided by Prof. Patrick van Damme, were grown in a greenhouse at typical winter day/night temperature regime of 19/8 °C and light intensity, at noon, about 250 μmol·m^−2^·s^−1^ PPFD (Photosynthetic Photon Flux Density). The seeds were germinated, within 4 to 6 days, on top of saturated potting soil and covered by another 2 cm layer of dry soil mixed with Osmocote Plus (Scotts Miracle-Gro, Scotts Australia, Bella Vista, Australia). About 25% of the seeds germinated after 3 days with an approximate 1:1 ratio of male:female. It took approximately 4 to 6 weeks for the plants to begin flowering.

### 4.2. Experimental Design

The morphological, physiological and anti-oxidant systems of female and male *M. annua* plants were compared along their development, from the ‘young’ (55 days) to ‘late senescence’ (122 days), with and without salt (NaCl).

Establishing a reference time course scale of development of female and male plants was done by growing the plants in soil and harvesting three plants of each gender every two weeks before noon, beginning from ’young’ until the ‘late senescence’ stage. Fully developed leaves were collected, halved along the main vein of the leaf, for assay of the enzymes and substrates on the same leaf in each plant and stored separately at −80 °C for assays of the anti-oxidative enzymes, APX, CAT, POD and SOD and substrates, ASC, H_2_O_2_ and MDA.

The effect of salinity on female and male plants was conducted using ‘mature’ (84 days) plants grown in salinity for 11 days as follows: 32 plants (16 females and 16 males) of *M. annua* gown in soil were uprooted at day 84 (‘mature stage’) from sowing and transferred to 16 L containers containing aerated-half-strength Hoagland solution, with four female and four male plants per container. Two of the containers were used as control without NaCl and two for salinity treatment challenged with 100 mM NaCl. Three days after transfer to the hydroponic system, salt treatment began by the addition of 33.3 mM salt, with subsequent 33.3 mM daily incremental increases up to 100 mM NaCl. After eleven days in 100 mM NaCl, the leaves from the top 5th node were harvested, cut in half along the main vein, and stored at −80 °C for analysis. All samples were assayed for the anti-oxidative enzymes, APX, CAT, POD and SOD and substrates, ASC, H_2_O_2_ and MDA. In the case of the GST activity ‘early senescence’ plants (98 days) were exposed to NaCl (150 mM) for 2 days.

### 4.3. CO_2_/H_2_O Gas Exchange

Photosynthetic, transpiration rates and stomatal conductance were measured in control and NaCl (100 mM) treated plants. In the experiment of the long term effect of salinity, plants were grown under salinity from one week after germination until the gas exchange measurements were conducted at days 42 and 59 after sowing. In the short term salinity effect experiment, 56 days old plants grown in soil without salt (control) were transferred to the salinity treatment (NaCl 100 mM) and the gas exchange measurements were conducted after 2 h and 3 days. In both experiments, 4 plants (of each gender) were analyzed in control (no salinity) and 8 plants in salinity treatments. For each sampling time, five measurements were made on each plant. Measurements were done using a portable gas-exchange measuring system (GFS-3000, H. Walz GmbH, Effeltrich, Germany), at a CO_2_ concentration of 380 μmol·mol^−1^, 25 °C and a photosynthetic photon flux density of 250 μmol·m^−2^·s^−1^.

### 4.4. Protein Extraction

Total soluble proteins for anti-oxidative enzyme assays were extracted by grinding 1–2 g of plant material in a mortar and pestle in the presence of liquid nitrogen. Extraction was conducted according to Mittova *et al.* (2000) [60]. The extraction procedure for GST detoxification enzymes was carried out according to Schröder *et al.* (2005) [61]. Protein content of the samples was measured according to Bradford (1976) [62] with bovine serum albumin as a standard protein.

### 4.5. Enzyme Assays

Ascorbate peroxidase (APX, EC 1.11.1.11) activity was determined immediately after the extraction by monitoring the decrease in optical density at 290 nm, according to Jiménez *et al.* (1997) [63].

Catalase (CAT, EC 1.11.1.6) activity was followed spectrophotometrically by the decrease in absorption at 240 nm, resulting from H_2_O_2_ consumption [64].

Superoxide dismutase (SOD, EC 1.15.1.1.) activity was determined by monitoring the inhibition of photochemical reduction of nitro-blue-tetrazolium (NBT) at 560 nm, according to Beyer and Fridovich (1987) [65]. One unit of SOD activity was defined as the amount of protein required to cause 50% inhibition of NBT reduction.

Guaiacol peroxidase (POD, EC 1.11.1.9) activity was followed spectrophotometrically by the increase of absorption at 470 nm, resulting from tetra-guaiacol formation by enzymatic oxidation of guaiacol [66].

Glutathione S-transferase (GST, EC 2.5.1.18) activity was assayed spectrophotometrically by the method of Schröder *et al.* (2005) [61] using 1-chloro-2, 4- dinitrobenzene (CDNB, ε340 nm = 9.6 mM^−1^·cm^−1^), 1, 2- dichloro-4-nitrobenzene (DCNB, ε345 nm = 8.5 mM^−1^·cm^−1^), 4-nitrobenzyl chloride (NBC, ε310 nm = 1.8 mM^−1^·cm^−1^) and p-nitrobenzoyl chloride (NBoC, ε310 nm = 1.9 mM^−1^·cm^−1^), respectively, as substrates. Assays using the herbicide fluorodifen (ε400 nm = 3.1 mM^−1^·cm^−1^) as a substrate followed Scalla and Roulet (2002) [67].

### 4.6. Anti-Oxidative Metabolites Extraction and Assay

Reduced ascorbate content was measured spectrophotometrically via the reduction of Fe^3+^ to Fe^2+^ by ASC and was monitored at 525 nm [68].

Lipid peroxidation was estimated by assessment of malondialdehyde (MDA) using the method of Draper & Hadley (1990) [69].

Hydrogen peroxide content was assayed according to Wolff (1994) [70].

### 4.7. Metabolite Profiling and NMR Identification

Plants of *M. annua* at ‘young stage’ (55 days) were exposed to NaCl (100 mM) for 3 days. Extraction of polar metabolites from female and maleleaf and root was done with perchloric acid, based on the protocol by Bouzier *et al.* (2000) [71]. In brief, plant material was extracted by 1 M perchloric acid. The mixture was centrifuged, the supernatant was neutralized with 1M KOH, and the pellet was dissolved in 700 μL of D_2_O. The solution was centrifuged. To the supernatant (550 μL), 3-(trimethylsilyl)-1-propanesulfonic acid was added to a final concentration of 0.19 mM as internal standard. ^1^H NMR was recorded at 500 MHz using a Bruker DRX 500 instrument equipped with an inverse ^1^H/^13^C probehead (300 K, 64 K data set; 45° pulse-angle, 4 μs; spectral width, 5.5 kHz; 64 scans; water suppression by pre-saturation). NMR signals were tentatively assigned using Chenomx NMR suite 4.6.

## 5. Statistical Analysis

Statistical analysis was done using SigmaPlot 2000 Windows Version 6.10. Significance analysis comparing responses of female *vs.* male plants and organs (leaf *vs.* root) during development were conducted by Student *T*-test. One Way Anova—Fisher’s test was used in order to compare the specific activity of enzymes, metabolites and/or groups of metabolites in relation to gender, leaf, root and salinity. In the experiments involving enzymes, substrates and metabolites the data represents averages of at least 3 plants (indicated by *n* = 3 in the legends to figures and tables), each analyzed in triplicates.

## Figures and Tables

**Table 1:** Morphological parameters of female and male *M. annua* plants during development.

Age (Days *)	Plant Height (cm)		Root Length (cm)		Reproductive Node (No.)	
	male	female	male	female	male	female
55 (‘young’)	25 ± 5 ^a^	25 ± 2 ^a^	5 ± 1 ^a^	5 ± 1 ^a^	5 ± 0 ^a^	5 ± 0 ^a^
70 (‘early maturity’)	40 ± 4 ^b^	38 ± 2 ^b^	6 ± 4 ^a,b^	6 ± 1 ^a,b^	7 ± 1 ^b^	7 ± 1 ^b^
84 (‘mature stage‘)	48 ± 0 ^c^	48 ± 3 ^c^	8 ± 0 ^b^	7 ± 1 ^b^	7 ± 1 ^b^	6 ± 1 ^a,b^
98 (‘early senescence’)	60 ± 3 ^d^	61 ± 3 ^d^	8 ± 1 ^b^	8 ± 1 ^b^	6 ± 1 ^a,b^	7 ± 1 ^b^
114 (‘senescence‘)	61 ± 5 ^d^	54 ± 5 ^d^	12 ± 5 ^c^	13 ± 1 ^c^	6 ± 1 ^a,b^	12 ± 1 ^c^
122 (‘late senescence’)	77 ± 16 ^d^	58 ± 11 ^d^	14 ± 5 ^b,c^	9 ± 3 ^b,c^	8 ± 1 ^b^	11 ± 1 ^c^

* Days from sowing. Plant length is of the above ground main stem. Nodes were counted on the main stem only. Values are Ave ± SD, *n* = 3. Significantly different values in a column (*p* < 0.05) are assigned different letters.

**Table 2:** Morphological parameters of female and male *M. annua* plants under salinity treatment.

Treatment	Gender	Fresh Weight (gr)	Plant Height (cm)	Root Length (cm)	Reproductive Node (No.)
Control	Male	20 ± 7 ^a^	63 ± 7 ^b^	13 ± 7 ^c^	9 ± 1 ^d^
Salinity	Female	23 ± 11 ^a^	56 ± 10 ^b^	17 ± 5 ^c^	9 ± 1 ^d^
	Male	18 ± 6 ^a^	59 ± 9 ^b^	13 ± 3 ^c^	7 ± 2 ^e^
	Female	20 ± 8 ^a^	54 ± 7 ^b^	14 ± 4 ^c^	12 ± 1 ^f^

Plants at the ‘mature stage’ (day 84) were grown for additional 11 days in the presence or absence of NaCl (100 mM). *n* = 8; Ave ± SD; Significantly different values (*p* < 0.05) within a column are assigned different letters. Weight and length are of above ground organs. For plant appearance see Figure S4.

## Supplementary Materials

The following are available online at http://www.mdpi.com/2218-1989/6/2/13/s1, Figure S1: Developmental stages of *M. annua*. Figure S2: Short term effect of salinity on physiological parameters. Figure S3: Long term effect of salinity on physiological parameters. Figure S4: ‘Mature stage’ *M. annua* plants under salinity treatment. Figure S5: Ratio of APX, CAT and POD activities to SOD activity in leaves of *M. annua* during development. Figure S6: Effect of salinity on metabolites’ concentration in *M. annua* leaf and root. Table S1: GST activity in female and male *M. annua* leaves under salinity. Table S2: Analysis of variance of metabolites concentration in female and male leaf and root of *M. annua* under salinity.

## Abbreviations

APX	Ascorbate peroxidase
ASC	Ascorbate
CAT	Catalase
DMA	dimethylamine
GST	Glutathione S-transferase
MDA	Malondialdehyde
NBT	Nitro-blue-tetrazolium
POD	Guaiacol Peroxidase
ROS	Reactive Oxygen Species
SOD	Superoxide dismutase
TCA	Tricarboxylic Acid Cycle
