# Supplementary material for: Sexual Dimorphism in the Response of Mercurialis annua to Stress
Source: Metabolites. 2016 Apr 26;6(2):13. doi: 10.3390/metabo6020013 (PMC4931544; doi:10.3390/metabo6020013)
Supplement: Supplementary file 1 [file metabolites-06-00013-s001.pdf]

Supplementary Materials: Sexual Dimorphism in the Response of *Mercurialis annua* to Stress

Ezra M. Orlofsky, Giorgi Kozhoridze, Lyudmila Lyubenova, Elena Ostrozhenkova, J. Barbro Winkler, Peter Schröder, Adelbert Bacher, Wolfgang Eisenreich, Micha Guy and Avi Golan-Goldhirsh

Table S1. GST activity in female and male *M. annua* leaves under salinity.

Specific Activity (μmol Product mg·protein^{-1}·sec^{-1})		
	Control	Salinity
Female	4.12 ^{a} ± 0.40	0.80 ^{c} ± 0.23
Male	0.83 ^{b} ± 0.11	1.14 ^{c} ± 0.13

GST activity using fluorodifen as substrate. Plants at 'early senescence stage' were grown in medium containing 150 mM NaCl, for 48 h. Values are Ave ± SD, $n = 3$. Different superscript letters in a row indicate significant difference at $p < 0.05$.

Table S2. Analysis of variance of metabolites concentration in female and male leaf and root of *M. annua* under salinity.

Interaction	Notes	Grouping order	P	DF
RC*RS	Male and Female	All metabolites	0.27	114
LC*LS			0.85	114
LC*RC			0.00	114
LC*RS			0.00	114
LS*RC			0.02	114
LS*RS			0.06	114
M*F	Male and Female, Salinity and Control		0.97	230
L*R			0.00	230
ML*FL	Salinity and control		0.85	114
ML*FR			0.00	114
MR*FL			0.02	114
MR*FR			0.46	114
AA F* AA M	Root and Leaf, Salinity and Control	Groups of metabolites	0.6	44
CF * CM			0.4	14
TCA F* TCA M			0.75	70
SM F*SM M			0.04	22
AA RC*AA RS	Male and Female		0.75	22
C RC*C RS			0.47	6
TCA RC*TCA RS			0.87	34
S RC*S RS			0.8	12
AA LC*AA LS			0.9	22
C LC*C LS			0.5	6
TCA LC*TCA LS			0.37	34
S LC*S LS			0.8	12

Statistical analyses of metabolites concentrations were determined by ^{1}H NMR (see Figure S5). Plant organs of 'young stage' plants of *M. annua* grown for 4 days under 100 mM NaCl, were analyzed. Analysis of variance was done on all metabolites and groups of metabolites as detailed in the notes column. Abbreviations: RC—Root Control, RS—Root salinity, LC—Leaf Control, LS—Leaf salinity, M—Male, F—Female, L—Leaf, R—Root, ML—Male Leaf, FL—Female Leaf, FR—Female Root, MR—Male Root, AA F—Amino Acids in Female, AA M—Amino acids in Male, CF—Carbohydrates in Female, CM—Carbohydrates in Male, TCA F—TCA metabolites in Female, TCA M—TCA cycle metabolites in Male, SM F—Secondary Metabolites in Female, SM M—Secondary Metabolites in Male,

AA RC—Amino Acids in Root Control, AA RS—Amino Acids in Root salinity, C RC—Carbohydrates in Root Control, C RS—Carbohydrates in Root salinity, TCA RC—TCA metabolites in Root Control, TCA RS—TCA metabolites in Root salinity, S RC—Secondary Metabolites in Root Control, S RS—Secondary Metabolites in Root salinity, AA LC—Amino Acids in Leaf Control, AA LS—Amino Acids in Leaf salinity, C LC—Carbohydrates in Leaf Control, C LS—Carbohydrates in Leaf salinity, TCA LC—TCA Metabolites in Leaf Control, TCA LS—TCA Metabolites in Leaf salinity, S LC—Secondary Metabolites in Leaf Control, S LS—Secondary Metabolites in Leaf salinity, DF = degree of freedom.

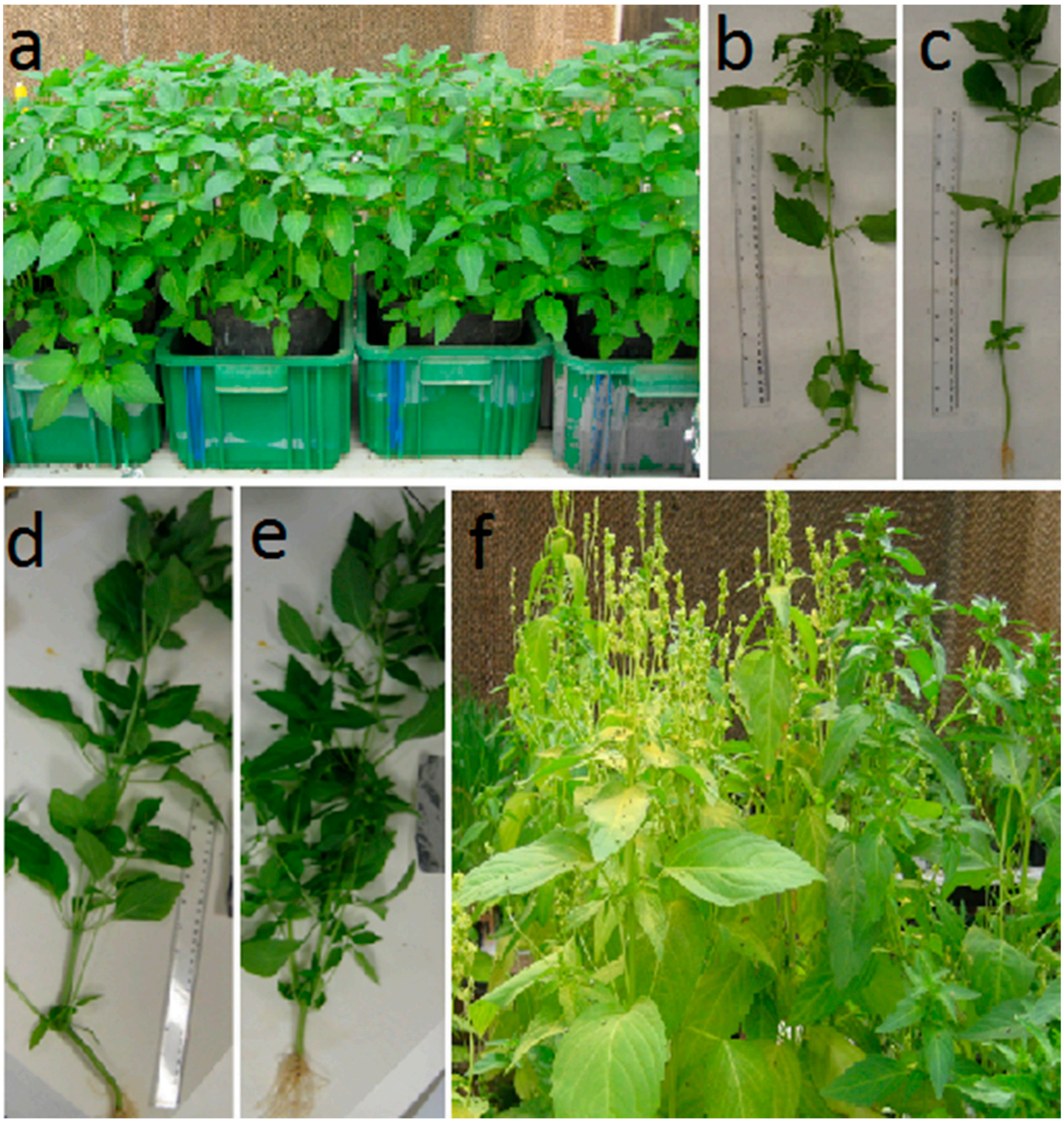

Figure S1. Developmental stages of *M. annua* (**a**): 'young' plants at day 55; (**b**) and (**c**): 'early Maturity stage' male and female, respectively at day 70; (**d**) and (**e**): 'mature stage' female and male, respectively, at day 84; (**f**): 'early senescence stage' day 98 males (on **left**) and females (on **right**). Note that female plants were greener and male plants were covered by yellow pollen grains.

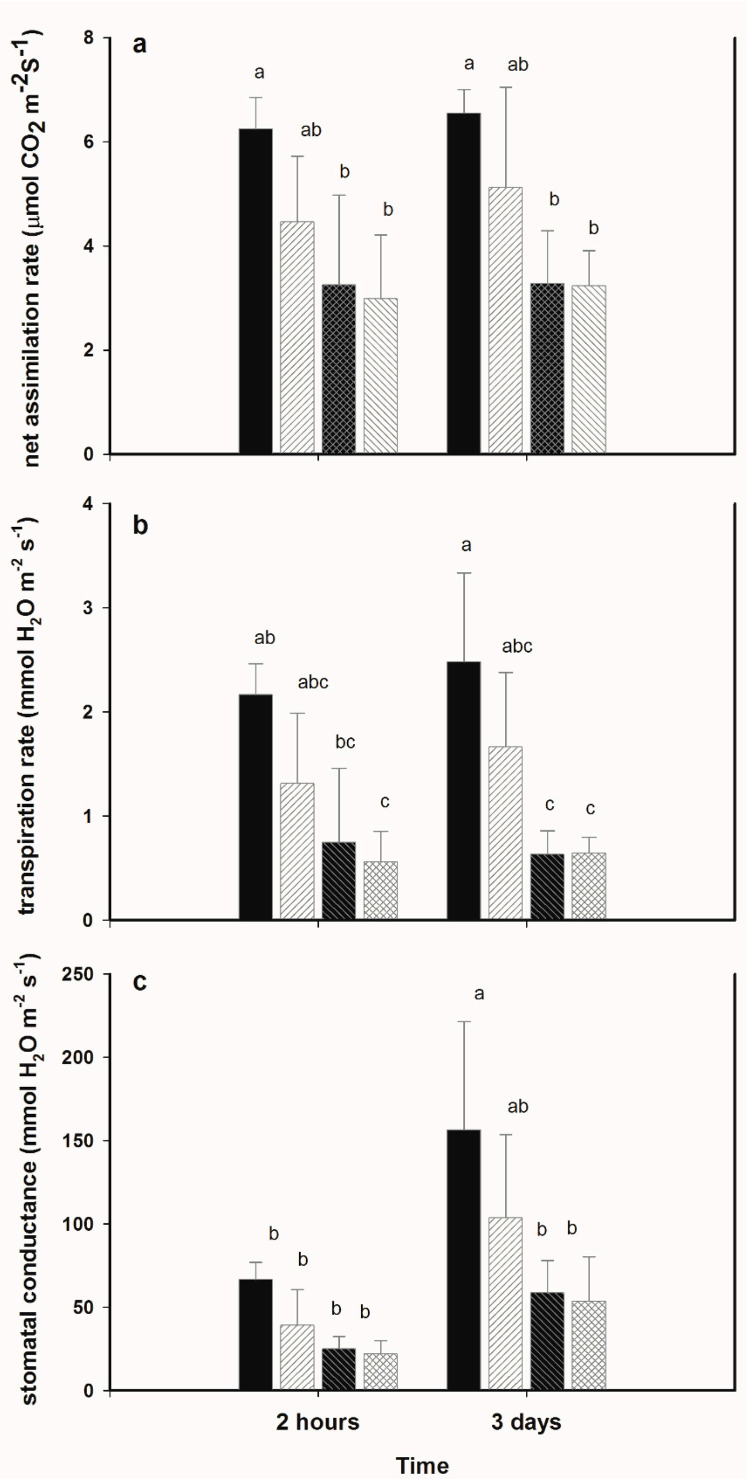

Figure S2. Short term effect of salinity on physiological parameters. (**a**) Net CO_2 assimilation rate; (**b**) Transpiration rate; (**c**) Stomatal conductance. Fifty six days old plants grown in soil without salt (control) were transferred to the salinity treatment (NaCl, 100 mM) and the gas exchange measurements were conducted after 2 h and 3 days. ■—female control; ▨—male control; ▧—female salinity; ▩—male salinity.

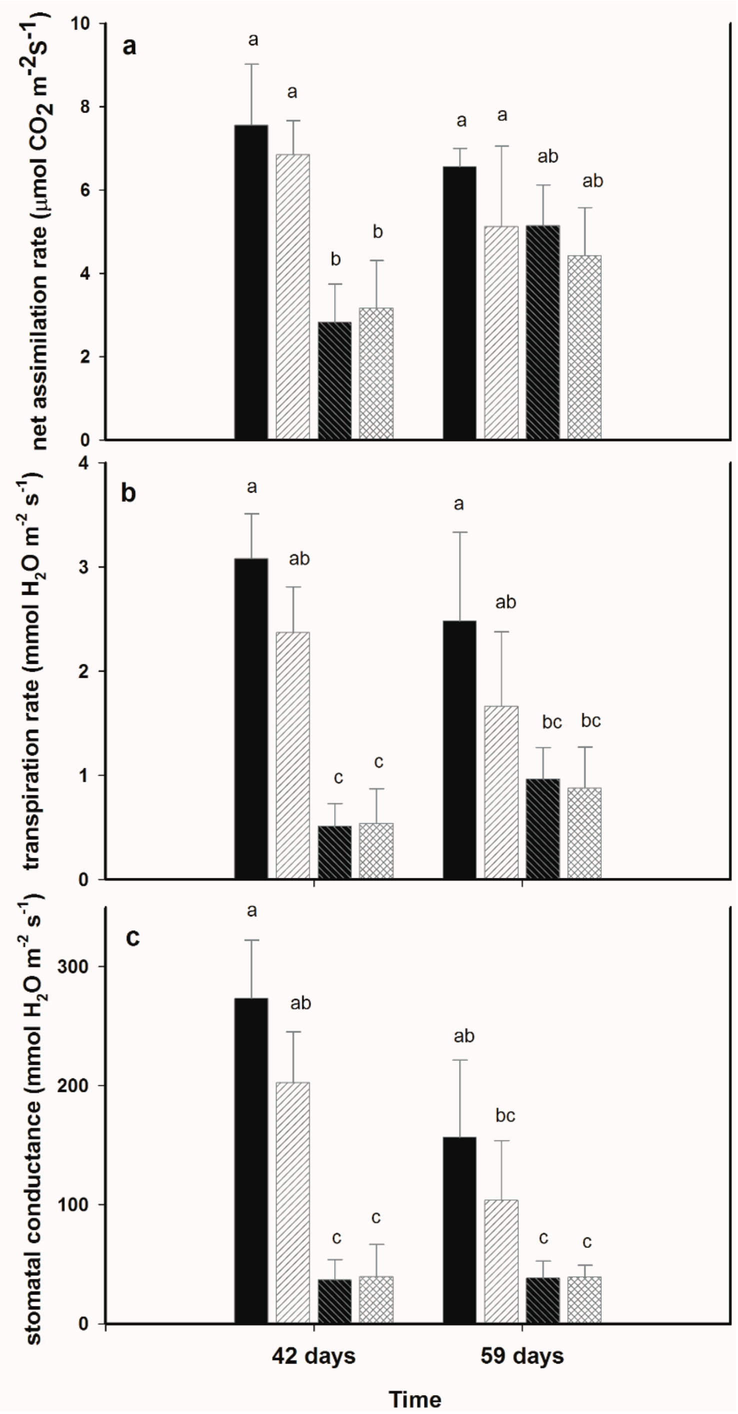

Figure S3. Long term effect of salinity on physiological parameters. (**a**) Net CO_2 assimilation rate; (**b**) Transpiration rate; (**c**) Stomatal conductance. Plants were grown under salinity (NaCl, 100 mM) from one week after germination until the gas exchange measurements were conducted at days 42 and 59 after sowing. ■—female control; ▨—male control; ▩—female salinity; ▦—male salinity.

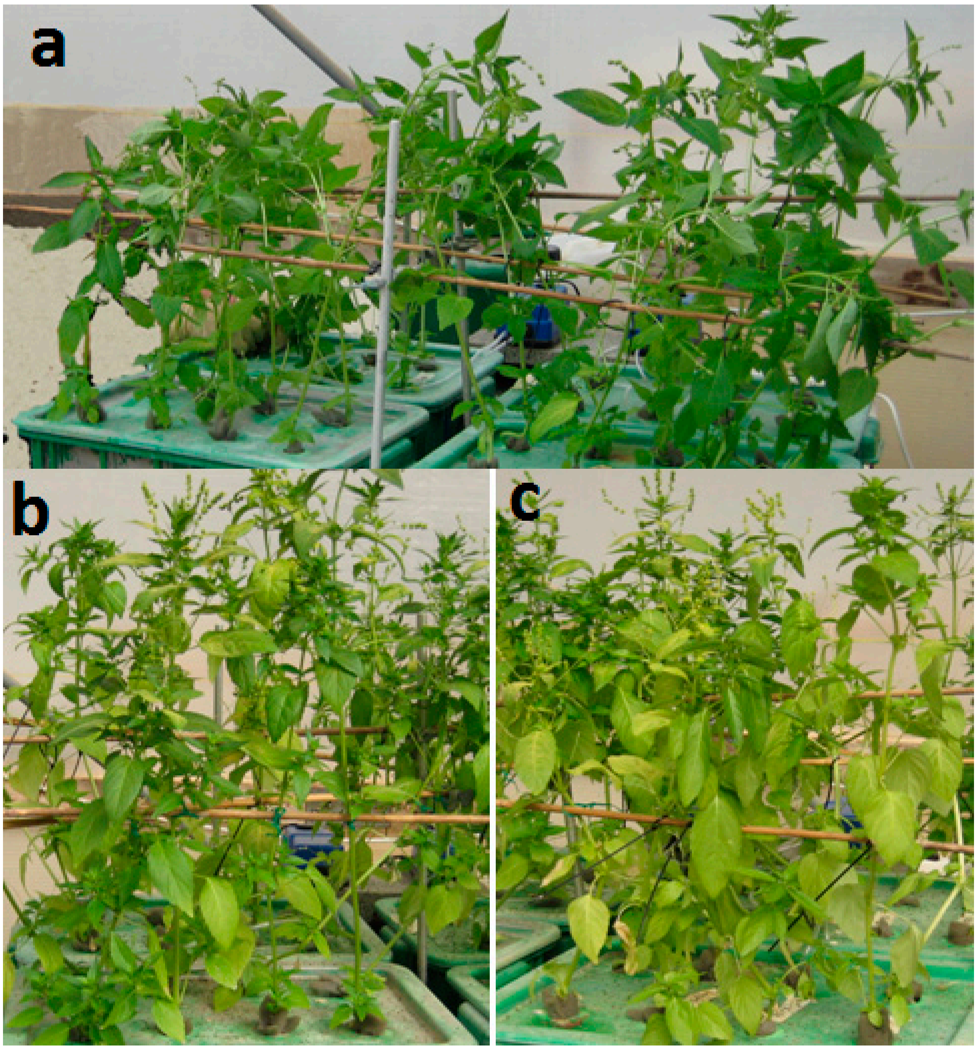

Figure S4. 'Mature stage' *M. annua* plants under salinity treatment. (**a**): At the beginning of the experiment; (**b**) and (**c**): Control and NaCl (100 mM) treated plants for 11 days, respectively.

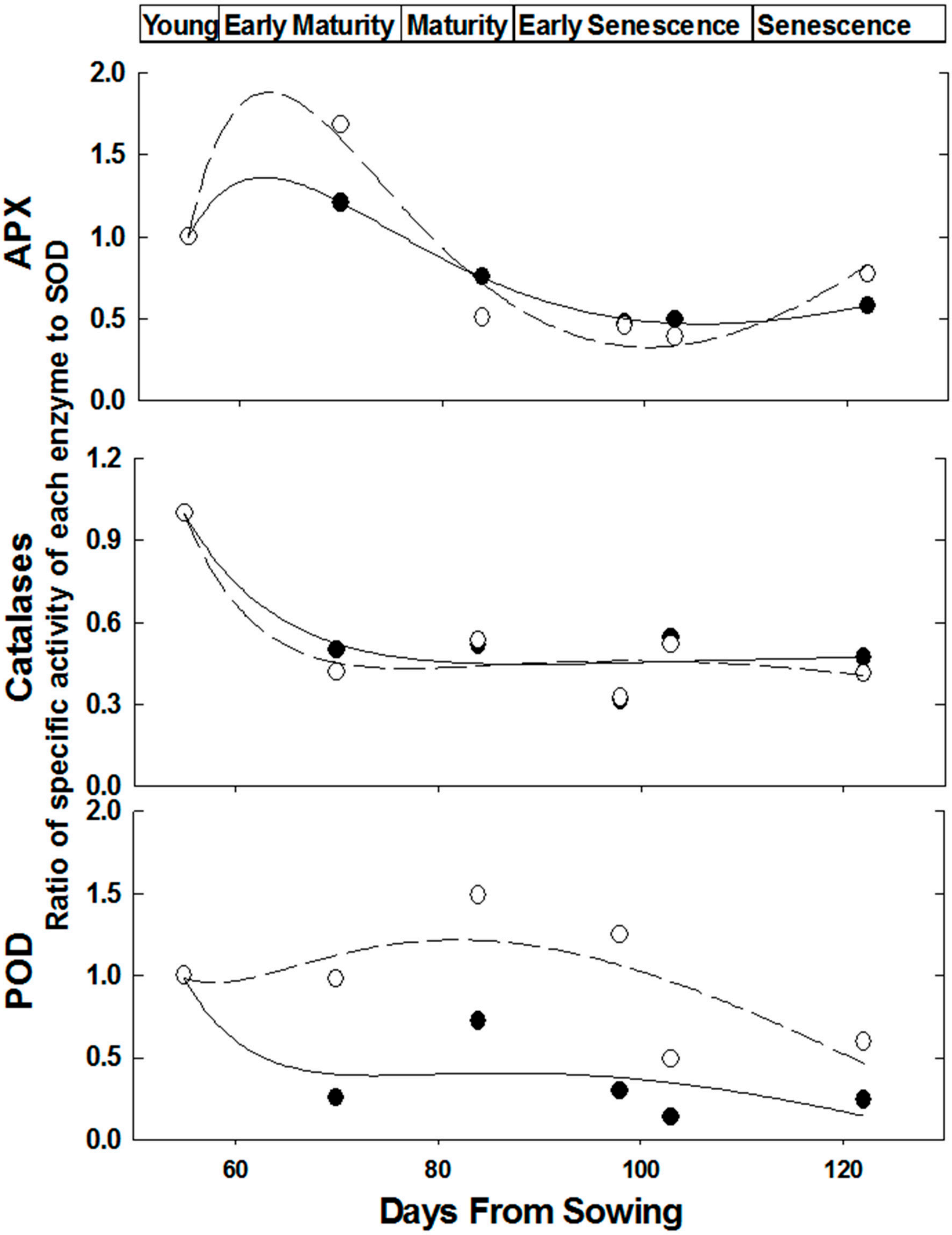

Figure S5. Ratio of APX, CAT and POD activities to SOD activity in leaves of *M. annua* during development. The ratio of each H_2O_2 quenching enzyme activity to SOD activity at day 55 was assigned 1. The data were fitted by a 3rd order polynomial curve. Ave ± SD, *n* = 3. Open circle—female plant; Black circle—male plant.

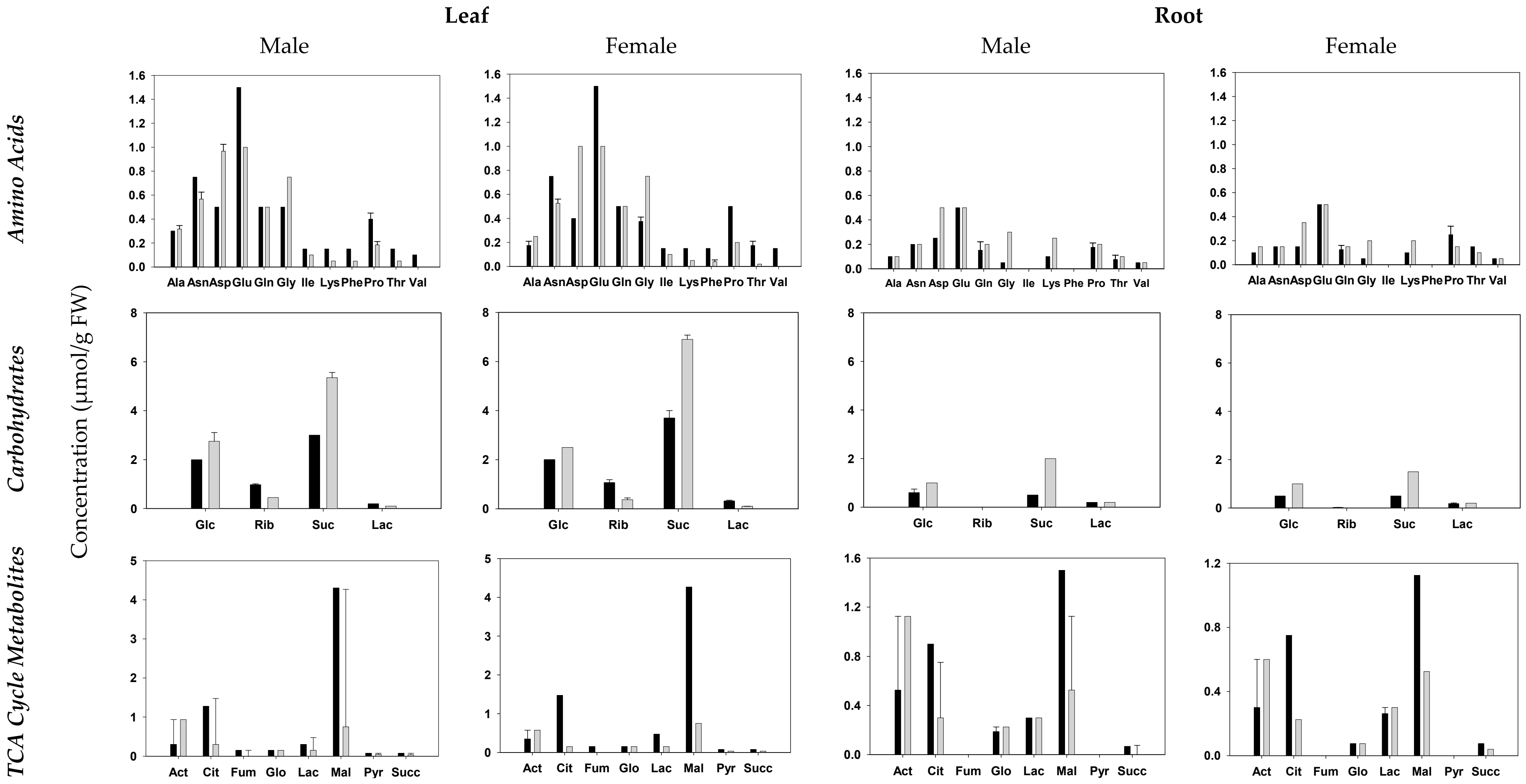
Leaf
Root
Male
Female
Male
Female
Amino Acids
Carbohydrates
TCA Cycle Metabolites
Concentration (µmol/g FW)
Ala Asn Asp Glu Gln Gly Ile Lys Phe Pro Thr Val
Glc Rib Suc Lac
Act Cit Fum Glo Lac Mal Pyr Succ

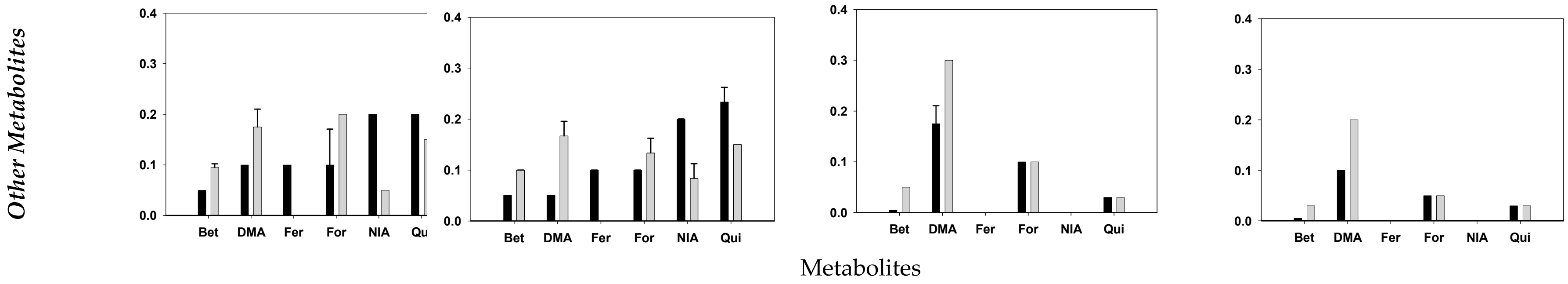

Figure S6. Effect of salinity on metabolites' concentration in *M. annua* leaf and root.
